# Effects of MRI scanner manufacturers in classification tasks with deep learning models

**DOI:** 10.1038/s41598-023-43715-5

**Published:** 2023-10-05

**Authors:** Rafsanjany Kushol, Pedram Parnianpour, Alan H. Wilman, Sanjay Kalra, Yee-Hong Yang

**Affiliations:** 1 Department of Computing Science, University of Alberta, Edmonton, Canada; 2 Neuroscience and Mental Health Institute, University of Alberta, Edmonton, Canada; 3 Departments of Radiology and Diagnostic Imaging and Biomedical Engineering, University of Alberta, Edmonton, Canada; 4 Division of Neurology, Department of Medicine, University of Alberta, Edmonton, Canada

**Keywords:** Network models, Machine learning

## Abstract

Deep learning has become a leading subset of machine learning and has been successfully employed in diverse areas, ranging from natural language processing to medical image analysis. In medical imaging, researchers have progressively turned towards multi-center neuroimaging studies to address complex questions in neuroscience, leveraging larger sample sizes and aiming to enhance the accuracy of deep learning models. However, variations in image pixel/voxel characteristics can arise between centers due to factors including differences in magnetic resonance imaging scanners. Such variations create challenges, particularly inconsistent performance in machine learning-based approaches, often referred to as domain shift, where the trained models fail to achieve satisfactory or improved results when confronted with dissimilar test data. This study analyzes the performance of multiple disease classification tasks using multi-center MRI data obtained from three widely used scanner manufacturers (GE, Philips, and Siemens) across several deep learning-based networks. Furthermore, we investigate the efficacy of mitigating scanner vendor effects using ComBat-based harmonization techniques when applied to multi-center datasets of 3D structural MR images. Our experimental results reveal a substantial decline in classification performance when models trained on one type of scanner manufacturer are tested with data from different manufacturers. Moreover, despite applying ComBat-based harmonization, the harmonized images do not demonstrate any noticeable performance enhancement for disease classification tasks.

## Introduction

Machine learning (ML) is a mathematical method based on statistics by which a computer model is created to perform specific tasks by learning from existing data and has been applied in clinical applications for many years. A prominent ML branch known as deep learning (DL) builds models using layers of interconnected neurons to learn critical insights from existing data and to predict the outcome for new data. Unlike traditional ML methods, DL networks automate feature extraction and selection, making them user-friendly and more prevalent than classical ML techniques. Recent research has demonstrated that DL, particularly convolutional neural networks (CNNs), are an effective strategy for classifying, segmenting, and detecting objects of interest in medical images^1–4^.

Magnetic resonance imaging (MRI) is a versatile, non-invasive imaging modality offering exceptional resolution and contrast for analyzing soft tissue. MR images have useful therapeutic applications, including diagnostics, due to the varied appearance of organs, tissues, and pathology. Training a DL model requires sufficient training data (e.g., MR images, clinical scores) and/or their corresponding ground truth. The network uses the training data to adjust its internal parameters (up to many millions), allowing it to map from the input to the required ground truth. The robustness of the model is highly dependent on the inclusion of a large number of relevant samples in the training phase. During deployment, the trained model is applied to unseen samples, leveraging its learned parameters to formulate predictions. Hence, the practical efficacy of the DL framework depends on its successful generalization to unknown datasets.

The aggregation of multi-center large-scale MRI databases in recent brain research initiatives has provided crucial findings for comprehending the neurobiological aspects underlying brain functions. However, the considerable variations arising from distinct centers, originating from non-biological sources and introducing variability into the neuroimaging data, have hindered the coherent interpretation of reported results. While numerous earlier studies have evaluated various CNNs across diverse MRI datasets, the generalization issue of CNNs on MR images remains. CNNs, as statistical tools, learn the input data’s statistics under the assumption of identical independent distribution (IID). Under this assumption, a trained CNN model is expected to perform consistently on samples with similar or identical distributions. In the context of multi-center MRI datasets, MR images are prone to statistical shifts due to variations in scanner manufacturers and different image acquisition protocols^5^.

MRI scanner variations contribute to significant statistical changes, as specific MRI machines provide images with unique properties due to vendor-specific proprietary implementations. The majority of the MRI scans observed in publicly available large datasets come from three renowned manufacturers: General Electric (GE) Healthcare, Philips Medical Systems, and Siemens^6^. Previous investigations have unveiled the limited generalization capability of CNN models across MRI data derived from different manufacturers. Tian et al.^7^ introduced an MRI harmonization technique that addresses various site effects, including factors such as scanner manufacturer, scanner type, phase encoding direction, and the number of channels per coil. However, the authors reported that the scanner manufacturer factor is the most significant parameter generating the site effects. In another MRI radiomics feature-based study^8^, the authors observed higher sensitivity in the scanner manufacturer parameter among the three scanner attributes (manufacturer, magnetic field strength, and slice thickness).

MRI has been widely used for three decades to diagnose diseases such as acute infarct^9^, multiple sclerosis (MS)^10^, brain tumors^11^ and so on. In a left ventricle (LV) segmentation task, the authors explored the performance variation among three different scanner manufacturers and proposed a manufacturer-adaptation strategy to mitigate scanner bias^12^. Dadar et al.^13^ assessed the reliability of gray and white matter volume measurements and the associated variability within multi-site MRI datasets utilizing different scanners. In another investigation^14^, the authors revealed a variability of 0.15 mm in cortical thickness measurements due to a scanner vendor change (GE/Siemens). However, to the best of our knowledge, no reports have been published demonstrating the impact on the performance of different disease classification tasks, such as patient vs. control normal (CN), due to variations in scanner manufacturers.

Harmonization is a technique used to mitigate variations arising from diverse image acquisition protocols. Multi-center MRI data harmonization aims to remove site-specific bias while preserving intrinsic image properties^15^ (e.g., biological factors). A popular harmonization method, “ComBat,” was initially introduced to alleviate batch effects in gene expression microarray data and has been proven effective in addressing scanner/site effects in multi-site diffusion tensor imaging (DTI) data^16^. We aim to evaluate the effectiveness of the standard ComBat and one of the modified ComBat-based harmonization approaches for structural imaging using four large multi-center longitudinal MRI datasets involving three major scanner manufacturers. Existing research shows that ComBat is highly successful in neuroimaging data harmonization, focusing on removing scanner effects from a set of imaging features such as cortical thickness, surface area, and subcortical volumes^17–20^. Pomponio et al.^21^ applied a modified ComBat method to 145 anatomical ROI volumes to eliminate location and scale effects for each ROI. Another study reported better performance by employing radiomic features from lung computed tomography (CT) images with a modified ComBat method^22^. Nevertheless, the application of a ComBat-based strategy to full-size 3D (NIFTI) images, rather than specific ROIs or extracted features, presents an ongoing challenge. For extensive high-resolution image datasets, memory allocation constraints may impede program execution. Additionally, the ComBat-based strategy requires some demographic data to be available for all samples, such as sex, age, and disease status, which we aim to preserve during harmonization. Importantly, adding a new sample to an existing dataset imposes another concern: the need to rerun the entire harmonization process with the newly added data. Lastly, a recent study^23^ disclosed that existing statistical harmonization methods like ComBat failed to harmonize cortical thickness from multi-scanner MRI data.

This work focuses on the non-biological factors of variability in neuroimaging data due to transformations in MRI scanner manufacturers, which pose a barrier to the practical applications of DL algorithms in the medical domain. We highlight how the scanner vendor significantly impacts disease classification performance with multiple DL models. Our investigation delves into the classification of patients with four complex neurodegenerative disorders: Alzheimer’s disease (AD), Parkinson’s disease (PD), amyotrophic lateral sclerosis (ALS), and the intermediate stage of AD known as mild cognitive impairment (MCI). Our analysis demonstrates a drastic drop in classification accuracy when DL models are tested with data from a different scanner manufacturer. Subsequently, our experiments reveal that employing a ComBat-based harmonization technique could not yield discernible enhancements in classification performance when applied to a multi-center dataset of 3D structural MR images.

## Methods

### Datasets

The Health Research Ethics Board (HREB) at the University of Alberta approved the protocol presented in this study. The Alzheimer’s Disease Neuroimaging Initiative (ADNI)^24^ and the Parkinson Progression Marker Initiative (PPMI)^25^ represent two prominent and extensively studied publicly available datasets in the field of AD and PD detection, respectively. The ADNI protocol received authorization from the committee on human research at each participating center, with written informed consent provided by each participant. Additional information is accessible at adni.loni.usc.edu. The PPMI study was performed following the Declaration of Helsinki and Good Clinical Practice (GCP) policies, in addition to the approval of the local ethics committees of the participating centers. At the time of enrollment, each participant provided written informed consent for using their imaging and clinical data. More details can be found at http://www.ppmi-info.org/. The authors attained approval to use the ADNI and PPMI data in the present study. The Canadian ALS Neuroimaging Consortium (CALSNIC)^26^ is the only prospective, multi-center and multimodal longitudinal study of ALS using harmonized clinical and imaging protocols across its sites. The CALSNIC study was conducted with the approval of each participating site’s HREB, and informed consent was obtained from the participants. Our study leverages T1-weighted MR images, commonly used for standard structural imaging, acquired from three distinct MRI manufacturers (GE, Philips, and Siemens) across the aforementioned datasets. The acquisition orientation of all the MRI data used in our study is sagittal. We employ two versions of ADNI, ADNI1 and ADNI2, consisting of 1638 and 865 MRI scans, respectively. Additionally, our study enlists 528 samples from PPMI and 545 samples from the CALSNIC2 datasets. CALSNIC1 data were excluded from our experiments due to its comparably limited sample size as well as variations in MRI acquisition orientation. An insightful depiction of the demographic composition of our utilized datasets is presented in Table 1. Furthermore, Table 2 meticulously outlines the divergent scanning protocols linked to different scanner manufacturers.

**Table 1:** Demographic details of the ADNI1, ADNI2, PPMI, and CALSNIC2 datasets.

Dataset	Group	MRI scanner manufacturer					
		GE		Siemens		Philips	
		Sex	Age	Sex	Age	Sex	Age
		(M/F)	(Mean±Std)	(M/F)	(Mean±Std)	(M/F)	(Mean±Std)
ADNI1	AD	80/80	75.5 ± 7.7	80/80	75.0 ± 7.2	60/49	75.7 ± 7.0
	CN	80/80	75.1 ± 5.7	80/80	75.9 ± 5.9	109/67	75.4 ± 5.2
	MCI	150/100	75.3 ± 7.6	150/100	76.1 ± 7.0	150/63	75.9 ± 7.5
ADNI2	AD	62/41	75.0 ± 8.5	100/57	75.1 ± 7.8	48/58	74.5 ± 7.3
	CN	80/82	74.3 ± 5.9	100/57	74.0 ± 6.4	80/100	75.6 ± 6.4
PPMI	PD	83/40	61.6 ± 9.7	78/46	63.0 ± 9.8	70/37	61.6 ± 9.9
	CN	17/17	59.6 ± 13.3	72/35	59.6 ± 10.5	20/13	59.7 ± 11.2
CALSNIC2	ALS	14/4	54.0 ± 11.8	124/65	60.1 ± 10.2	29/20	62.4 ± 8.2
	CN	18/13	60.1 ± 8.8	120/101	54.9 ± 10.5	12/25	61.7 ± 10.8

**Table 2:** Scanning protocol details of the ADNI1, ADNI2, PPMI, and CALSNIC2 datasets.

Dataset	Scanning protocol	MRI scanner manufacturer		
		GE	Siemens	Philips
ADNI1	Model	Genesis Signa, Signa Excite,	Symphony, Sonata, Trio,	Achieva, Intera Achieva,
		Signa HDx	TrioTim, Avanto, Allegra	Intera, Gyroscan Intera
	Field strength	1.5 T / 3.0 T	1.5 T / 3.0 T	1.5 T / 3.0 T
	Flip angle	8 °	8 °/ 9 °	8 °
	Spatial resolution	$1.0\times 1.0\times 1.2$ ${\rm mm}^3$ /	$1.0\times 1.0\times 1.2$ ${\rm mm}^3$ /	$1.0\times 1.0\times 1.2$ ${\rm mm}^3$ /
		$0.94\times 0.94\times 1.2$ ${\rm mm}^3$	$1.25\times 1.25\times 1.2$ ${\rm mm}^3$	$0.94\times 0.94\times 1.2$ ${\rm mm}^3$
ADNI2	Model	Signa HDxt, Signa Excite,	Symphony, Skyra, Verio,	Achieva dStream, Achieva,
		Signa HDx, Discovery MR750	TrioTim, Avanto	Intera, Ingenia, Ingenuity
	Field strength	3.0 T	3.0 T	3.0 T
	Flip angle	11 °	9 °	9 °
	Spatial resolution	$1.05\times 1.05\times 1.2$ ${\rm mm}^3$	$1.05\times 1.05\times 1.2$ ${\rm mm}^3$	$1.05\times 1.05\times 1.2$ ${\rm mm}^3$
PPMI	Model	Signa HDxt, Signa Excite,	Symphony, Skyra, Verio,	Achieva dStream, Intera,
		Discovery MR750w, Genesis Signa	TrioTim, Prisma, Espree,	Achieva, Gyroscan NT
		Signa Architect, Discovery MR750	Prisma Fit	
	Field strength	1.5 T / 3.0 T	1.5 T / 3.0 T	1.5 T / 3.0 T
	Flip angle	8 °/ 11 °/ 13 °/ 15 °	8 °/ 9 °/ 15 °	8 °/ 9 °
	Spatial resolution	$1.0\times 1.0\times 1.0$ ${{\rm mm}}^3$ /	$1.0\times 1.0\times 1.0$ ${{\rm mm}}^3$ /	$1.0\times 1.0\times 1.0$ ${\rm mm}^3$ /
		$0.94\times 0.94\times 1.2$ $\textrm{mm}^3$/	$1.25\times 1.25\times 1.3$ $\textrm{mm}^3$/	$0.94\times 0.94\times 1.2$ $\textrm{mm}^3$/
		$0.94\times 0.94\times 0.7$ ${\rm mm}^3$	$0.49\times 0.49\times 2.0$ ${\rm mm}^3$	$1.0\times 1.0\times 1.2$ $\textrm{{ mm}}^3$
CALSNIC2	Model	Discovery MR750	Prisma, Prisma Fit, TrioTim	Achieva
	Field strength	3.0 T	3.0 T	3.0 T
	Flip angle	16 °	10 °	10 °
	Spatial resolution	$1.0\times 1.0\times 1.0$ ${\rm mm}^3$	$1.0\times 1.0\times 1.0$ ${\rm mm}^3$	$1.0\times 1.0\times 1.0$ ${\rm mm}^3$

### Preprocessing

A straightforward, rapid, and commonly employed preprocessing pipeline is implemented to prepare the original 3D T1-weighted brain MRI data for disease classification tasks. The process begins with a standard operation known as skull stripping, aimed at eliminating the unnecessary skull region. This task is achieved using the *FreeSurfer* program^27^ (Command: mri_synthstrip -i input_image -o stripped_image)^3^. Subsequently, we perform N4 bias field correction using the *SimpleITK* library’s N4BiasFieldCorrectionImageFilter class to rectify low-frequency intensity non-uniformity in the MRI data^28^. The Symmetric normalization (SyN) registration technique, implemented through *ANTsPy*^29^, is then employed to align each scan with the MNI-152 standard space, utilizing lanczosWindowedSinc interpolation for transformation. Lastly, we apply *WhiteStripe* intensity normalization using the Python intensity-normalization package^30^. Upon completing the preprocessing of the original images, their dimensions are transformed to $182\times 218\times 182$, and the voxel size is converted to $1\times 1\times 1$ $mm^3$. This preprocessing procedure typically takes around 5 minutes per scan, with computations performed on an eight-core CPU platform utilizing parallel processing.

### DL models

**Figure 1:**
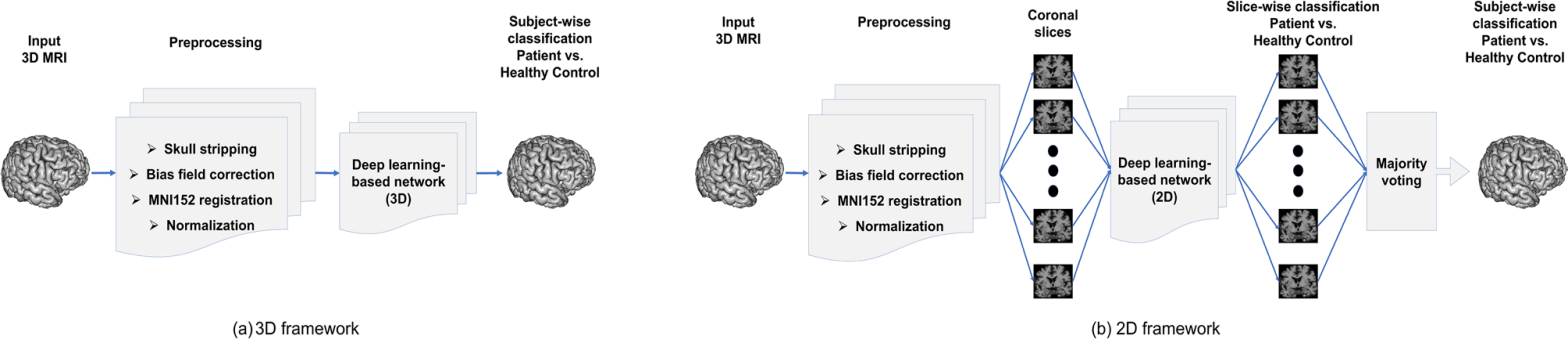
The processing pipeline used in our study to carry out different disease classification tasks with different DL networks.

To assess the performance of various classification tasks across datasets with distinct scanner vendors, we employ both 2D and 3D DL architectures. Firstly, we utilize three widely recognized and successful networks: ResNet^31^, ShuffleNetV2^32^, and MobileNetV2^33^. Subsequently, we employ two customized models designed explicitly for AD classification. The Residual Network (ResNet), a prominent and influential DL model, was introduced by He et al.^31^. A pivotal contribution of ResNet is the introduction of “identity shortcut connections,” creating alternate pathways for gradient flow and addressing the vanishing gradient problem in deep CNNs. The fundamental building block of MobileNet^33^ is depthwise separable convolution, which comprises depthwise convolution and pointwise convolution. Depthwise convolution applies distinct kernels to each input channel, while pointwise convolution employs $1 \times 1$ convolution kernels. ShuffleNet^32^, designed to accommodate mobile device computing limitations, relies on pointwise group convolution and channel shuffling to maintain accuracy while significantly reducing computational load. Qiu et al.^1^ introduced a 3D customized Fully Convolutional Network (FCN) consisting of six convolutional blocks and then integrated both neuroimaging and clinical data using Multilayer Perceptron (MLP) networks. However, our study only employs their FCN model to handle neuroimaging data. Meanwhile, ADDFormer^4^, inspired by the vision transformer (ViT) architecture^34^, combines frequency and spatial domain features in an innovative manner. ADDFormer employs selected coronal 2D slices, and leverages transfer learning by pre-training the network on ImageNet^35^. Figure 1 illustrates the processing pipeline for both 2D and 3D frameworks. In the case of 3D networks, after preprocessing, DL models analyze the entire 3D brain MRI data to extract features for the final class prediction. Conversely, for 2D networks, we assess 15 coronal slices from the central position for feature extraction. The final classification decision is determined by majority voting of class predictions from these coronal slices of a subject, similar to the approach used in ADDFormer.

## Results

### Experimental setup

The DL frameworks employed in our analysis are implemented using PyTorch^36^ and executed on a server equipped with 4 NVIDIA RTX A6000 GPUs. The coding of 3D CNN models is based on publicly available implementations, accessible at https://github.com/xmuyzz/3D-CNN-PyTorch. To enhance training robustness, we employ data augmentation methodologies, including random rotations, flipping, and the mixture of Gaussian noise, to prepare a robust training batch. The optimization process employs the Adam optimizer with an initial learning rate of 0.00005 and a decay rate of $10^{-1}$ after every 100 iterations. For the ADDFormer model, a patch size of $16 \times 16$ is used, and the training spans a total of 300 epochs with a batch size of 16. The final accuracy reported in this study represents the average results from five experiments, each employing distinct training, validation, and test data combinations. The data split ratio is maintained at 70% for training, 15% for validation, and 15% for testing in each experimental setup. The training time of the CNN-based procedures takes approximately 6 hours on a single GPU with 48GB of memory. The classification performance is evaluated using standard statistical metrics, specifically Accuracy (Acc) and F1-score. They are characterized in terms of four key values: True Positive (TP), True Negative (TN), False Positive (FP), and False Negative (FN). The *Acc* metric represents the fraction of accurately identified subjects to the total number of samples in a given dataset, defined as $Acc = \frac{TP + TN}{TP + TN + FP + FN}$. The *F1-score* harmonically combines precision and recall, and is mathematically measured as *F1-score* $= 2 \times \frac{precision \times recall}{precision + recall}$. The recall is the ability to identify individuals with a specific condition correctly and is computed as $recall = \frac{TP}{TP + FN}$. The precision reflects the number of relevant items and can be expressed as $precision = \frac{TP}{TP + FP}$.

### Scanner manufacturer effects

**Figure 2:**
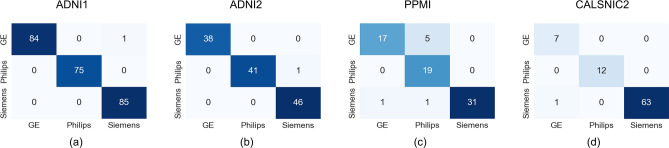
MRI scanner manufacturer classification results for the ADNI1, ADNI2, PPMI, and CALSNIC2 datasets generated by ResNet model. The classification accuracy is approximately 99% for the (**a**) ADNI1, (**b**) ADNI2, and (**d**) CALSNIC2 datasets whereas the accuracy is around 95% for the (**c**) PPMI dataset.

**Figure 3:**
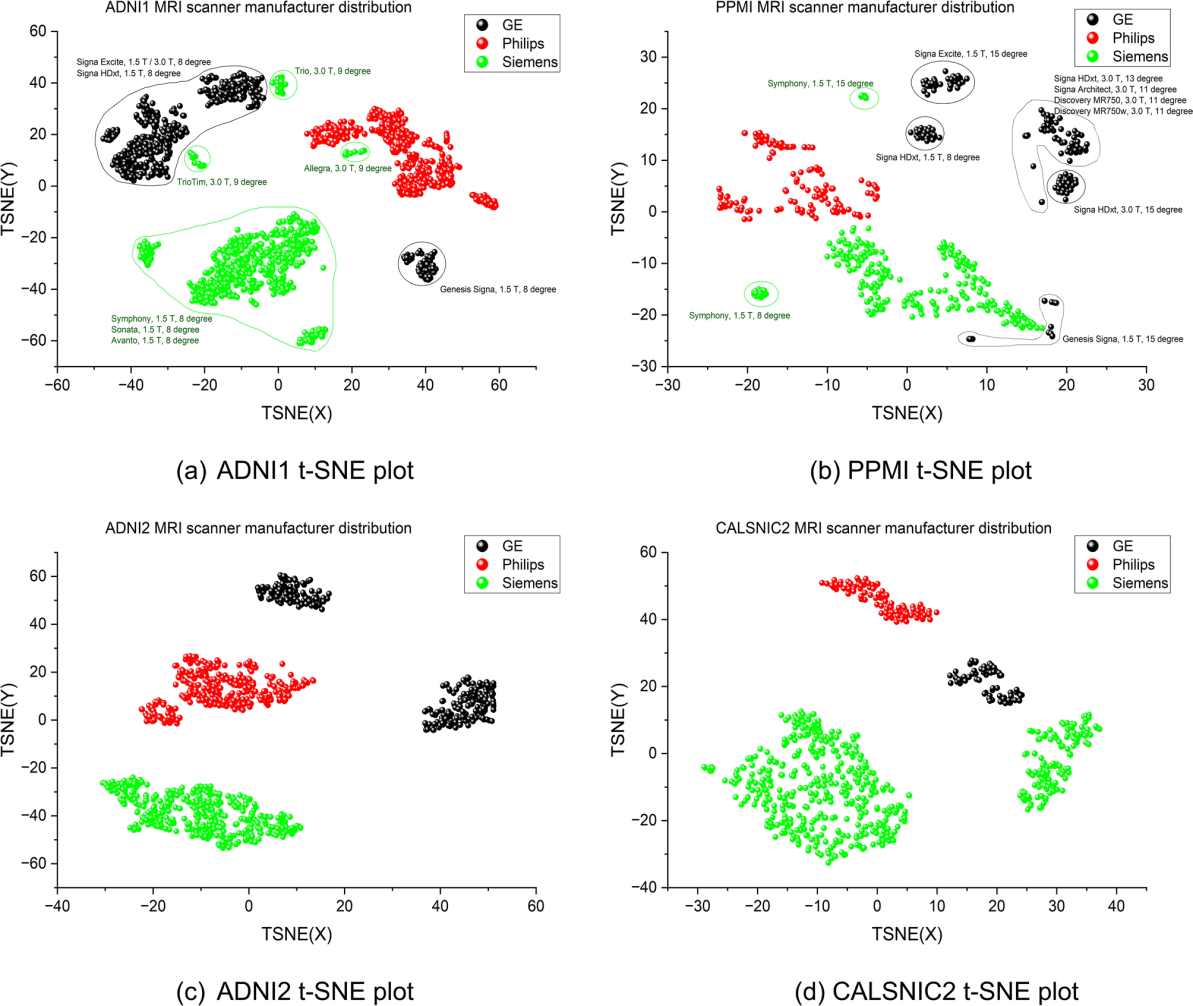
t-SNE plots for the ADNI1, ADNI2, PPMI, and CALSNIC2 datasets using the features generated by MRQy evaluation metrics. Different clusters are primarily formed based on the scanner manufacturer. In panels (**a**) and (**b**), bounding boxes are delineated, incorporating information about the scanner model, field strength, and flip angle. These annotations visually highlight their role in inducing domain shift within a dataset.

This section presents the results of a series of experiments highlighting the distinctive characteristics of different scanner manufacturers. Initially, we employ three 3D DL-based classification networks (ResNet^31^, MobileNetV2^33^, ShuffleNetV2^32^) using MRI data to classify three distinct scanner manufacturers (GE, Philips, and Siemens). These well-established CNN-based networks demonstrate exceptional accuracy in classifying the scanner manufacturers. For the ADNI1, ADNI2, and CALSNIC2 datasets, the average classification accuracy exceeds 98%, while the accuracy for the PPMI database ranges between 93% and 96% across all the aforementioned frameworks. The classification outcomes, presented as confusion matrices derived from the ResNet architecture for different datasets, are depicted in Fig. 2. The corresponding confusion matrices for the ShuffleNetV2 and MobileNetV2 models can be found in Supplementary Figs. S1 and S2, respectively. Subsequently, we employ t-SNE (t-distributed Stochastic Neighbor Embedding)^37^ and UMAP (Uniform Manifold Approximation and Projection)^38^ techniques to visualize the data in a 2D space, using features generated by MRQy^39^. These visualizations are presented in Fig. 3 and Supplementary Fig. S3. Both t-SNE and UMAP are non-linear, graph-based dimension reduction methods that project the high-dimensional feature space into a lower-dimensional space while preserving the distribution characteristics. The visualization of the t-SNE and UMAP plots reveals that the proximity of grouped data primarily corresponds to the scanner manufacturer. Additionally, we observe further clustering within the same vendor, which can be attributed to variations in scanner models from the same manufacturer. Minor contributions to data clustering arise from variations in magnetic field strength and flip angles, as depicted by different bounding boxes in Fig. 3. The 3D views of the t-SNE and UMAP plots are available on our GitHub project page at https://github.com/rkushol/Effects-of-MRI-scanner-manufacturer.

### Gender classification

The task of gender classification (Male vs. Female) from MRI data is comparatively less intricate than the challenge of classifying different neurodegenerative diseases. In this context, we evaluate gender classification across the four previously mentioned datasets to assess performance variations among different scanner manufacturers. The outcomes of gender classification, achieved through distinct 3D CNN-based deep models (ResNet^31^, MobileNetV2^33^, ShuffleNetV2^32^), are presented in Table 3. For the ADNI1, ADNI2, and CALSNIC2 datasets, the aforementioned CNN methods achieve an average accuracy and F1-score of over 90%. Notably, in the PPMI dataset, using data from Siemens and GE also yields an average accuracy of around 90%, while using Philips data results in an approximate classification accuracy of 85%. Overall, there is no significant difference in performance among the scanner manufacturers in this classification task.

**Table 3:** Gender classification results for the ADNI1, ADNI2, PPMI, and CALSNIC2 datasets.

Scanner manufacturer	DL models	ADNI1		ADNI2		PPMI		CALSNIC2	
		Acc	F1-score	Acc	F1-score	Acc	F1-score	Acc	F1-score
GE	ResNet	0.92	0.93	0.93	0.94	0.93	0.92	0.92	0.92
	ShuffleNetV2	0.95	0.94	0.92	0.93	0.89	0.90	0.96	0.96
	MobileNetV2	0.92	0.93	0.91	0.90	0.88	0.89	0.92	0.92
Siemens	ResNet	0.94	0.94	0.92	0.91	0.90	0.90	0.91	0.90
	ShuffleNetV2	0.94	0.93	0.97	0.95	0.94	0.92	0.92	0.92
	MobileNetV2	0.90	0.89	0.91	0.90	0.88	0.88	0.90	0.91
Philips	ResNet	0.92	0.91	0.90	0.90	0.86	0.87	0.95	0.94
	ShuffleNetV2	0.93	0.93	0.90	0.88	0.85	0.84	0.94	0.94
	MobileNetV2	0.90	0.89	0.92	0.93	0.84	0.83	0.93	0.92

### Disease classification

**Figure 4:**
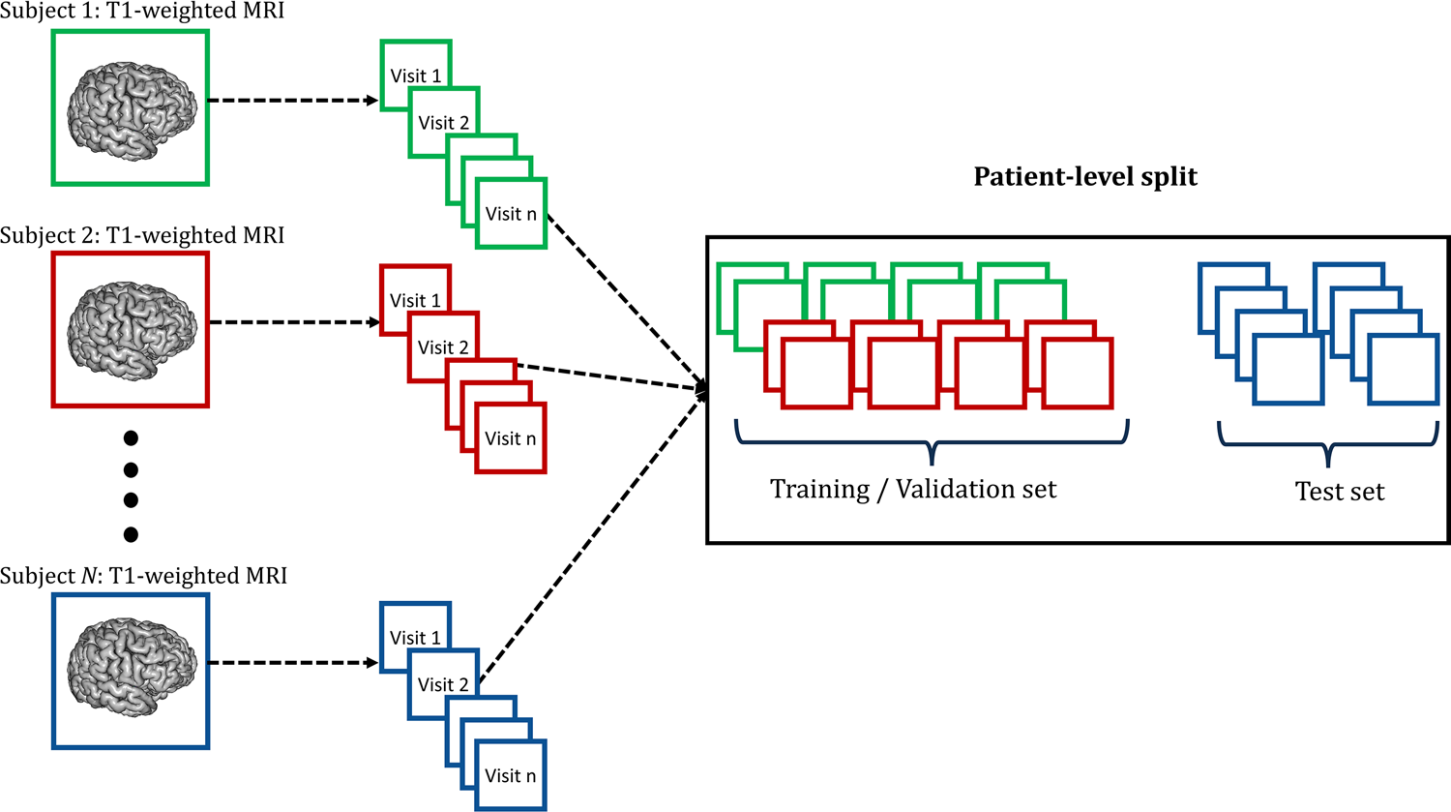
Patient-level split process for longitudinal data to train different DL models.

Classifying patients with neurodegenerative diseases such as AD, PD, or ALS from healthy controls using limited MRI data poses significant challenges due to the subtle structural changes present in the images. To enhance the reliability of our findings while maintaining balanced sample sizes across different scanner manufacturers, we leverage longitudinal data. However, a notable exception arises in the CALSNIC2 dataset, where the volume of data from GE and Philips scanners is comparatively smaller compared to that of the Siemens vendor. Moreover, we ensure that our data-splitting strategy avoids data leakage issues. This involves meticulously dividing the data based on individual subjects, preventing mixing the same participant’s images in both training and testing processes, as illustrated in Fig. 4. In the context of 2D frameworks, we extend this practice to ensure the integrity of slices within subjects across the test and training sets. Indeed, a recent study^40^ discovered that many prior disease classification approaches did not follow a proper distribution of slices or subjects in their training or testing data. As a result, their reported outcomes present inaccurate and excessively optimistic classification accuracies. Our analysis reveals that the ResNet (3D) and FCN (3D) models outperform other 3D frameworks across various disease classification tasks. Similarly, in the case of 2D networks, the ResNet (2D) and ADDFormer (2D) models achieve better results compared to other 2D DL methods. Table 4 summarizes the classification results from these top-performing models. The classification outcomes of the remaining four DL techniques are also provided in Supplementary Table S1.

**Table 4:** Different disease classification results based on scanner manufacturers with the ADNI1, ADNI2, PPMI, and CALSNIC2 datasets.

Scanner manufacturer	DL models	AD vs. CN				MCI vs. CN		PD vs. CN		ALS vs. CN	
		ADNI1		ADNI2		ADNI1		PPMI		CALSNIC2	
		Acc	F1-score	Acc	F1-score	Acc	F1-score	Acc	F1-score	Acc	F1-score
GE	ResNet (3D)	0.76	0.76	0.79	0.79	0.70	0.68	0.80	0.80	0.70	0.70
	FCN^1^ (3D)	0.84	0.83	0.84	0.83	0.74	0.74	0.84	0.83	0.75	0.74
	ResNet (2D)	0.81	0.79	0.81	0.81	0.71	0.70	0.79	0.79	0.71	0.70
	ADDFormer^4^ (2D)	0.86	0.85	0.89	0.88	0.75	0.73	0.88	0.87	0.82	0.79
Siemens	ResNet (3D)	0.77	0.78	0.80	0.82	0.71	0.71	0.66	0.66	0.71	0.72
	FCN^1^ (3D)	0.84	0.84	0.82	0.82	0.73	0.73	0.70	0.71	0.75	0.76
	ResNet (2D)	0.78	0.76	0.76	0.74	0.71	0.70	0.66	0.66	0.71	0.69
	ADDFormer^4^ (2D)	0.88	0.88	0.86	0.85	0.71	0.72	0.72	0.71	0.78	0.79
Philips	ResNet (3D)	0.75	0.73	0.83	0.82	0.66	0.66	0.77	0.76	0.70	0.69
	FCN^1^ (3D)	0.84	0.83	0.86	0.85	0.71	0.70	0.80	0.80	0.73	0.72
	ResNet (2D)	0.74	0.73	0.79	0.78	0.67	0.66	0.74	0.73	0.70	0.67
	ADDFormer^4^ (2D)	0.85	0.85	0.91	0.90	0.71	0.71	0.82	0.82	0.79	0.79
All samples (GE + Siemens + Philips)	ResNet (3D)	0.76	0.78	0.79	0.80	0.71	0.69	0.76	0.77	0.72	0.72
	FCN^1^ (3D)	0.84	0.85	0.85	0.84	0.77	0.75	0.78	0.78	0.74	0.75
	ResNet (2D)	0.77	0.76	0.78	0.78	0.72	0.71	0.73	0.72	0.72	0.70
	ADDFormer^4^ (2D)	0.88	0.88	0.89	0.89	0.76	0.75	0.80	0.79	0.81	0.81
One-third samples (GE + Siemens + Philips)	ResNet (3D)	0.72	0.70	0.78	0.77	0.66	0.68	0.73	0.74	0.67	0.67
	FCN^1^ (3D)	0.80	0.80	0.80	0.81	0.70	0.68	0.74	0.74	0.71	0.70
	ResNet (2D)	0.74	0.72	0.75	0.75	0.66	0.65	0.70	0.70	0.67	0.68
	ADDFormer^4^ (2D)	0.79	0.80	0.80	0.79	0.68	0.68	0.75	0.74	0.74	0.75

#### ADNI1

Firstly, the independent evaluation of AD classification performance across the three manufacturers yields very close accuracy results. The classification accuracy of the top-performing model falls within the range of 85%-88%. Secondly, comparable accuracy is achieved when combining data from all manufacturers, resulting in a sample size approximately three times larger than that of each individual vendor. However, when equalizing the total sample size to that of a single manufacturer (approximately one-third of the total samples), a noticeable decline in performance is observed. Thirdly, among the 3D frameworks, the customized FCN model achieves the highest score, while the ADDFormer model outperforms all others in terms of classification accuracy. On the other hand, a similar conclusion is depicted for the intermediate stage of AD, known as the MCI vs. CN classification task, except that the overall accuracy decreases from all angles.

#### ADNI2

The classification accuracy of ADNI2 slightly surpasses that of ADNI1. Among the three manufacturers, utilizing data from Philips scanners yields slightly better performance compared to data from GE or Siemens. The range of the best model’s classification accuracy falls between 86% and 91%. Upon merging data from all manufacturers, which increases the sample size to approximately three times that of individual vendors, the achieved accuracy remains consistent. However, performance experiences a noticeable decline when the sample size is reduced to that of a single manufacturer, accounting for roughly one-third of the total samples. Once again, among the 3D frameworks of DL models, both the ResNet and the custom-made FCN model achieve better results. In contrast, within the group of 2D methods, the ADDFormer model stands out for achieving the highest classification accuracy.

#### PPMI

In the PD vs. CN classification task, we initially added a few control samples from the ADNI2 dataset to ensure balanced sample sizes of patients and healthy controls across all three manufacturer groups, thus mitigating severe class imbalance issues. Notably, the FCN and ADDFormer custom-made models also demonstrate strong performance when compared to other fundamental CNN-based methods. The ShuffleNet achieves better outcomes in certain cases within the group of 3D frameworks. The range of the best model’s classification accuracy spans from 72% to 88%. Comparable classification results are observed whether the data originates from GE or Philips scanners. However, the outcomes using data from Siemens scanners are comparatively poor. This discrepancy could be due to sharing a small number of healthy control samples from the ADNI2 dataset, whereas the GE or Philips group shares a large number of control samples from the ADNI2. Likewise, employing a total sample size equivalent to that of an individual manufacturer (approximately one-third of the total samples) leads to a noticeable decline in performance.

#### CALSNIC2

The classification task involving ALS patients versus healthy controls within the CALSNIC2 database presents an even greater challenge compared to AD classification. All three manufacturers exhibit similar average classification accuracy. However, the performance of data originating from Siemens scanners is notably more reliable due to the inclusion of large samples from multiple centers. The range of the best model’s classification accuracy falls between 78% and 82%. The accuracy remains consistent when the data from all manufacturers are combined. Conversely, the performance experiences a noticeable decline when the sample size from the Siemens manufacturer is reduced to one-third. The number of scans from GE and Philips scanners remains unchanged, as their original sizes are already limited. Among the DL models in both 3D and 2D frameworks, the ADDFormer model once again stands out for its highest classification accuracy.

#### Cross-validation

This section examines the consequences of introducing a change in the test set data by employing a different manufacturer. The left panel of Table 5 illustrates the classification results for this cross-domain validation using the four top-performing DL models described earlier. In this experimental setup, data originating from a specific manufacturer is utilized as the training domain, while the remaining two serve as the test domains. When comparing these findings with the results presented in Table 4, it becomes evident that a significant drop in accuracy is observed across all datasets in Table 5. These outcomes further confirm the presence of a substantial domain shift inherent within the MRI data acquired from different manufacturers.

**Table 5:** The cross-domain intra-study disease classification accuracy before and after voxel-wise ComBat harmonization for the ADNI1, ADNI2, PPMI, and CALSNIC2 datasets.

Dataset	Training data	Testing data	Classification Acc with different DL models							
			Results before harmonization				Results after harmonization			
			ResNet	FCN	ResNet	ADDFormer	ResNet	FCN	ResNet	ADDFormer
			(3D)	(3D)	(2D)	(2D)	(3D)	(3D)	(2D)	(2D)
ADNI1 AD vs. CN	GE	Philips+Siemens	0.75	0.80	0.75	0.86	0.75	0.78	0.74	0.76
	Philips	GE+Siemens	0.69	0.74	0.68	0.71	**0.70**	0.72	**0.69**	**0.73**
	Siemens	GE+Philips	0.72	0.77	0.74	0.79	0.71	0.71	0.70	0.68
ADNI2 AD vs. CN	GE	Philips+Siemens	0.71	0.76	0.72	0.80	0.69	0.71	0.69	0.68
	Philips	GE+Siemens	0.71	0.74	0.71	0.75	0.63	0.65	0.66	0.63
	Siemens	GE+Philips	0.75	0.77	0.77	0.83	0.68	0.70	0.67	0.69
ADNI1 MCI vs. CN	GE	Philips+Siemens	0.66	0.71	0.66	0.71	0.64	0.67	**0.68**	0.70
	Philips	GE+Siemens	0.60	0.67	0.62	0.64	**0.61**	0.62	0.59	0.64
	Siemens	GE+Philips	0.65	0.67	0.64	0.66	0.65	0.64	0.63	0.65
PPMI PD vs. CN	GE	Philips+Siemens	0.62	0.63	0.60	0.63	0.60	0.62	0.59	0.56
	Philips	GE+Siemens	0.65	0.66	0.63	0.67	0.60	0.65	0.59	0.59
	Siemens	GE+Philips	0.56	0.61	0.60	0.60	**0.59**	**0.63**	**0.62**	**0.67**
CALSNIC2 ALS vs. CN	GE	Philips+Siemens	0.57	0.56	0.56	0.61	0.57	**0.57**	0.55	0.55
	Philips	GE+Siemens	0.59	0.59	0.60	0.62	0.56	0.58	**0.61**	0.62
	Siemens	GE+Philips	0.61	0.63	0.65	0.68	0.59	**0.65**	0.65	**0.71**

### ComBat harmonization effects

**Figure 5:**
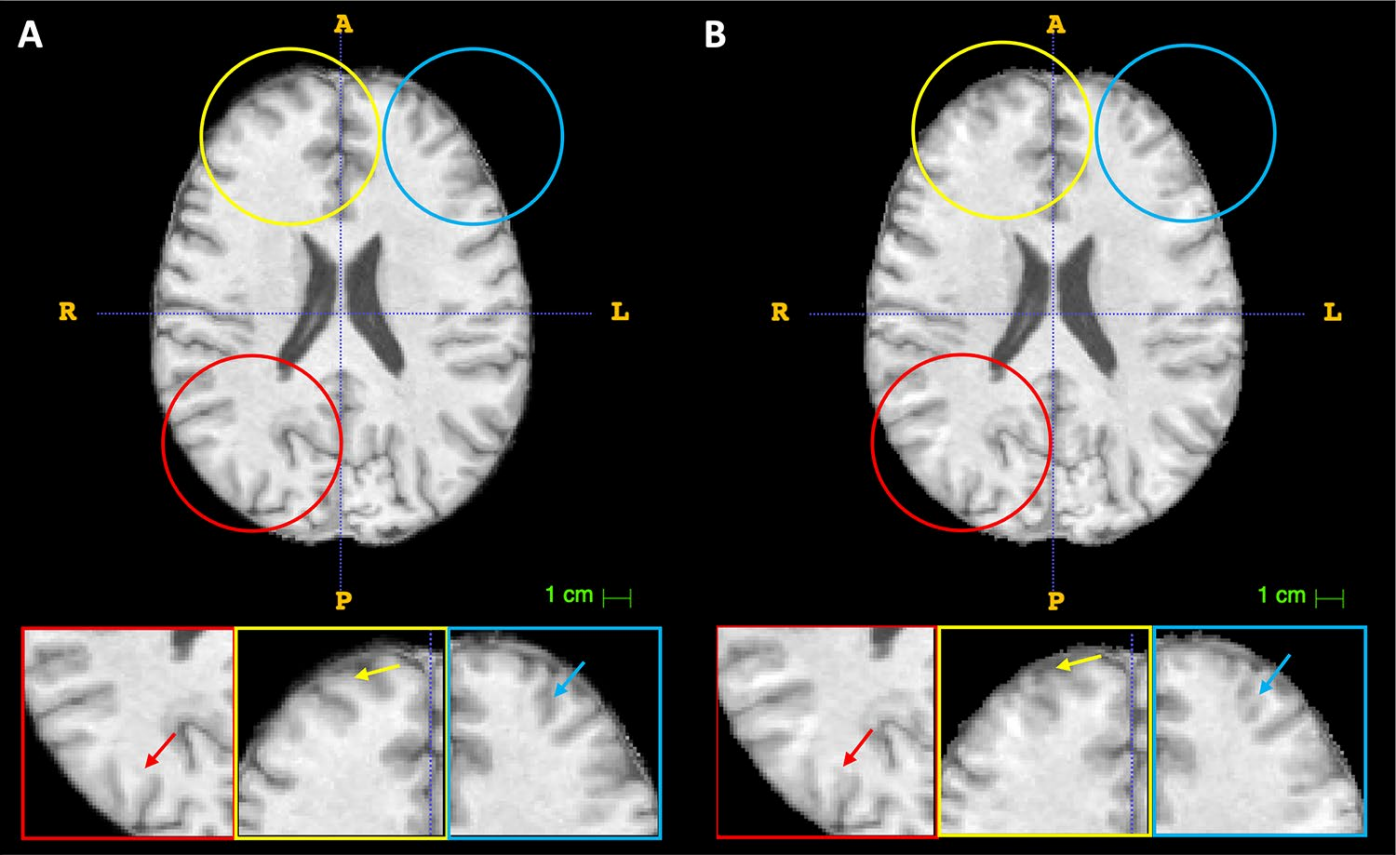
Minor changes in voxel-wise ComBat harmonization using structural MRI. (**A**) One 2D axial slice of preprocessed 3D T1-weighted MR image of CALSNIC2 dataset before harmonization, (**B**) corresponding slice after harmonization. The red, yellow, and blue arrows point to the regions with manipulated structures, including the disappearance of minor details resulting from the ComBat harmonization.

Initially, we evaluate the outcomes of a modified ComBat-based method known as ComBat-generalized additive model (ComBat-GAM), specifically designed to address site effects in multi-site neuroimaging datasets^21^. ComBat-GAM is the only publicly available package that directly handles 3D NIFTI images as input, accessible at https://github.com/rpomponio/neuroHarmonize. This technique successfully estimated age-related volume differences within a large-scale multi-center dataset, segmenting each MR image into 145 ROIs. However, our analysis does not yield promising outcomes when harmonizing entire 3D MRI data, as opposed to limited features extracted from MR images. Supplementary Fig. S4 provides an example of a 2D axial brain slice before and after harmonization using the ComBat-GAM method from the CALSNIC2 dataset. The output image exhibits undesirable artifacts and blurriness, with distinct brain tissue sections showing abnormal patterns of intensity shift compared to the input image. This disrupts the structural integrity of gray and white matter. As a result, we abstain from performing classification tasks using these undesirable resultant images generated by the ComBat-GAM approach. Subsequently, we apply the standard ComBat method to our multi-center datasets, utilizing the official implementation available at https://github.com/Jfortin1/ComBatHarmonization. A minor adjustment is made to the original implementation to enable voxel-level harmonization instead of feature-level harmonization, treating each scanner manufacturer as an individual site. From a visual perspective, the outcomes produced by the standard ComBat method closely resemble the original images, with minor changes evident in cortical regions, as depicted in Fig. 5. Thus, we harmonize our datasets using the standard ComBat and utilize the harmonized images for the cross-domain classification context. The classification results following the ComBat harmonization are presented in the right panel of Table 5. Unfortunately, the harmonized images generated by the standard ComBat method fail to enhance the classification accuracy in most cases (exceptions are shown in bold in Table 5). The potential reason behind these failures could be that ComBat-based harmonization techniques are inappropriate for image/voxel-level harmonization. Successful ComBat-based applications reported in prior studies have predominantly focused on limited feature-level harmonization. Moreover, during the execution of both ComBat-based strategies, we incorporate age and sex as covariates to ensure the preservation of this biological information throughout the harmonization process.

### Quality evaluation of scanner manufacturers data

Alongside manual inspection, we utilize the quality control tool MRQy^39^ to verify the quality of each MR image. The MRQy tool offers a comprehensive array of quality-related metrics, including peak signal-to-noise ratio (PSNR), contrast-to-noise ratio (CNR), coefficient of variation of the foreground patch (CVP) to address shading artifacts, coefficient of joint variation (CJV) to quantify aliasing and inhomogeneity artifacts between foreground and background, and entropy focus criterion (EFC) to detect motion artifacts. The user-friendly interface of MRQy greatly simplifies the process of identifying outliers or inconsistencies within a dataset. Table 6 presents an illustrative comparison of the diverse quality metrics obtained by averaging all samples for each scanner manufacturer.

**Table 6:** The quality evaluation of MRI data with MRQy for the ADNI1, ADNI2, PPMI, and CALSNIC2 datasets.

Dataset	Quality metrics	MRI scanner manufacturer		
		GE	Siemens	Philips
		(Mean ± Std)	(Mean ± Std)	(Mean ± Std)
ADNI1	PSNR $\uparrow$	15.69 ± 2.8	16.89 ± 1.1	**18.23 ± 1.6**
	CNR $\uparrow$	21.18 ± 9.0	19.41 ± 5.0	**50.16 ± 18.9**
	CVP $\downarrow$	**0.36 ± 0.1**	0.42 ± 0.1	0.41 ± 0.1
	CJV $\downarrow$	0.88 ± 0.2	**0.85 ± 0.1**	1.22 ± 0.3
	EFC $\downarrow$	**2.49 ± 0.4**	2.67 ± 0.2	2.60 ± 0.3
ADNI2	PSNR $\uparrow$	16.96 ± 1.0	15.37 ± 0.9	**17.16 ± 1.1**
	CNR $\uparrow$	17.64 ± 17.3	**34.09 ± 6.5**	12.31 ± 2.0
	CVP $\downarrow$	0.46 ± 0.1	**0.41 ± 0.1**	0.51 ± 0.1
	CJV $\downarrow$	1.59 ± 2.6	**0.91 ± 0.1**	1.48 ± 0.5
	EFC $\downarrow$	**1.89 ± 0.1**	2.94 ± 0.1	2.17 ± 0.2
PPMI	PSNR $\uparrow$	13.65 ± 1.5	14.42 ± 1.2	**17.31 ± 3.6**
	CNR $\uparrow$	29.29 ± 25.3	**34.99 ± 12.99**	16.33 ± 5.7
	CVP $\downarrow$	**0.39 ± 0.1**	0.41 ± 0.1	0.47 ± 0.1
	CJV $\downarrow$	**0.84 ± 0.2**	**0.84 ± 0.1**	0.95 ± 0.3
	EFC $\downarrow$	24.02 ± 13.1	4.04 ± 1.8	**3.36 ± 1.5**
CALSNIC2	PSNR $\uparrow$	**14.98 ± 0.9**	12.59 ± 1.6	11.82 ± 1.0
	CNR $\uparrow$	16.45 ± 4.3	**70.05 ± 24.8**	10.54 ± 2.4
	CVP $\downarrow$	0.41 ± 0.1	**0.37 ± 0.1**	0.46 ± 0.1
	CJV $\downarrow$	**0.72 ± 0.1**	0.84 ± 0.1	0.82 ± 0.1
	EFC $\downarrow$	10.1 ± 3.9	8.19 ± 3.0	**2.61 ± 0.2**

Best results are in [bold].

## Discussion

The reproducibility of MRI research continues to be challenging, particularly when data is influenced by scanner effects, a type of non-biological variation originating from various image acquisition protocols. After demonstrating significant distinguishable imaging characteristics present in data derived from multiple scanner manufacturers, we explore its consequences for different disease classification tasks using several prominent 2D and 3D DL models.

The primary challenge of this study was collecting an adequate number of MRI samples from three major scanner manufacturers (GE, Philips, and Siemens). The ADNI has satisfied our initial criterion, which offers the most extensive collection of publicly accessible research data resources, including imaging, clinical, and genomic data. In the ADNI1 and ADNI2 databases, the volume of data originating from Siemens and GE scanners was higher compared to that from Philips scanners. Therefore, we deliberately chose a comparable quantity of data from Siemens and GE vendors to match the data offered by Philips manufacturer. As we utilized longitudinal data, we prioritized including more unique subjects in this random selection scenario. In the context of the PPMI dataset, the data volume was more substantial for the Siemens scanners. Similarly, we limited the data from the Siemens manufacturer in a manner analogous to the sample size of GE and Philips scanners. The number of samples used in training an ML-based methodology, especially in DL models, plays a significant role in achieving satisfactory outcomes. Cohesive and consistent data enhance the performance in analysis, while the presence of heterogeneous characteristics in imaging data presents challenges in obtaining reliable and uniform results.

The preprocessing steps applied to our original T1-weighted MR images involve state-of-the-art algorithms and can be easily replicated using open-source tools. After experimenting with a straightforward classification task of differentiating sex (male vs. female) using the original MRI data, we move on to more sophisticated neurodegenerative disease classification tasks. Based on the results obtained from our applied DL models, the most challenging classification task is distinguishing between MCI and CN groups. This finding aligns with prior studies, which have also reported lower accuracy in this specific classification^41^. Notably, some investigations have further subdivided MCI into progressive (pMCI) and stable (sMCI) subgroups, achieving improved results through such stratification^42^. The next challenging task is the classification of PD vs. CN. One critical factor that makes this classification task difficult is the heterogeneous nature of the dataset. The PPMI dataset encompasses 21 different centers^25^, a characteristic evident in Fig. 3b. As a result, a decline in performance is anticipated in DL models if the test set contains data from a particular center, while the corresponding center’s data is either insufficient or entirely missing in the training set. For the same reason, tasks such as scanner vendor and gender classification might yield lower accuracy with the PPMI dataset compared to others. The classification task of distinguishing between ALS patients and healthy controls also presents challenges due to the insignificant structural changes in MRI data compared to the control group.

The specialized FCN model consistently outperformed other 3D classification frameworks in most disease classification scenarios. A notable advantage of 3D frameworks lies in their ability to process the entire brain as input, eliminating the need for prior knowledge in selecting specific slices for feature extraction. However, 3D DL methods tend to lack the utilization of pre-trained networks through transfer learning. In contrast, 2D frameworks necessitate the careful selection of relevant 2D slices based on prior knowledge. Additionally, the 2D DL models leverage the transfer learning property by utilizing pre-trained models with a massive 2D imaging dataset like ImageNet^35^. Overall, the ADDFormer network demonstrates the best performance in this study, leveraging the power of the ViT architecture by integrating spatial and frequency domain features in a novel manner. In fact, the process of capturing MRI scans initially involves representing data in the frequency domain before converting it to the spatial domain. Hence, effectively utilizing frequency domain features might be the key to achieving enhanced classification performance with MRI data. Furthermore, our study’s 2D models employed significant coronal slices related to disease pathology. The degeneration of nerve cells in brain regions such as the hippocampus, substantia nigra, and corticospinal tract, which are regarded as identifiable regions of interest in the pathogenesis of AD, PD, and ALS, respectively, were captured within the range of selected coronal slices given as input to the ADDFormer and other 2D models used in our study. A recent study similar to the ADDFormer network also demonstrates outstanding performance in the context of ALS classification^43^.

## Conclusion and future work

The field of neuroscience research requires robust, efficient, and reliable techniques to address the challenges posed by non-biological sources of data variation due to the increasing demand and necessity for multi-center neuroimaging studies. However, ML-based approaches have demonstrated limitations in producing consistent outcomes when confronted with data collected from diverse centers using distinct MRI scanner models and scanning protocols. Our experimental evaluation highlights the implications of incorporating MRI data from multiple manufacturers for disease classification tasks. Shifting the test domain with data from a different MRI vendor drastically drops the classification accuracy. Developing a novel framework for MRI data harmonization (adjusting scanner variability) becomes essential to effectively leverage multi-center neuroimaging studies. Domain adaptation methods have also emerged as a prominent research avenue in recent years, showing promising results in addressing domain shift and minimizing scanner-related biases. Exploring these solutions could offer valuable insights into refining the harmonization process and improving classification outcomes. Another exciting avenue for future work is analyzing the effects of different scanner models within the same scanner vendor. Last but not least, similar experiments could be conducted with multi-modal neuroimaging data such as FLAIR, functional MRI (fMRI), T2-weighted, and diffusion-weighted images to gain a comprehensive understanding of the effects of MRI scanner manufacturers.

### Supplementary Information

Supplementary Information.

## Data Availability

The neuroimaging data utilized in this study for the ADNI1, ADNI2, and PPMI were accessed through the ADNI portal at adni.loni.usc.edu. Acquisition of these datasets was facilitated through a standard application procedure. Additionally, the neuroimaging data from the CALSNIC2 database were curated and maintained by the Department of Medicine at the University of Alberta. Access to the CALSNIC2 data employed in our analysis can be requested by contacting kalra@ualberta.ca. Such requests will also be reviewed against alignment with established data-sharing protocols and privacy safeguards.
