# Supplementary material for: Effects of MRI scanner manufacturers in classification tasks with deep learning models
Source: Sci Rep. 2023 Oct 5;13:16791. doi: 10.1038/s41598-023-43715-5 (PMC10556074; doi:10.1038/s41598-023-43715-5)
Supplement: Supplementary file 1 — Supplementary Information. [file 41598_2023_43715_MOESM1_ESM.pdf]

Supplementary Information
Effects of MRI scanner manufacturers in classification tasks with deep learning models

Rafsanjany Kushol, Pedram Parnianpour, Alan H. Wilman, Sanjay Kalra, Yee-Hong Yang

Table S1: Different disease classification results based on scanner manufacturers with the ADNI1, ADNI2, PPMI, and CALSNIC2 datasets.

**Scanner manufacturer**	**DL models**	**AD vs. CN**				**MCI vs. CN**		**PD vs. CN**		**ALS vs. CN**	
		**ADNI1**		**ADNI2**		**ADNI1**		**PPMI**		**CALSNIC2**	
		**Acc**	**F1-score**	**Acc**	**F1-score**	**Acc**	**F1-score**	**Acc**	**F1-score**	**Acc**	**F1-score**
**GE**	ShuffleNetV2 (3D)	0.75	0.76	0.75	0.75	0.67	0.66	0.80	0.81	0.66	0.66
	MobileNetV2 (3D)	0.74	0.74	0.71	0.70	0.68	0.68	0.80	0.81	0.70	0.68
	ShuffleNetV2 (2D)	0.76	0.76	0.74	0.74	0.67	0.68	0.77	0.78	0.65	0.64
	MobileNetV2 (2D)	0.75	0.76	0.72	0.70	0.64	0.64	0.78	0.76	0.67	0.67
**Siemens**	ShuffleNetV2 (3D)	0.76	0.76	0.78	0.78	0.70	0.70	0.70	0.67	0.68	0.67
	MobileNetV2 (3D)	0.76	0.76	0.77	0.76	0.66	0.65	0.64	0.64	0.72	0.73
	ShuffleNetV2 (2D)	0.75	0.75	0.77	0.76	0.68	0.68	0.69	0.68	0.68	0.66
	MobileNetV2 (2D)	0.72	0.72	0.73	0.74	0.64	0.64	0.66	0.67	0.69	0.69
**Philips**	ShuffleNetV2 (3D)	0.76	0.76	0.83	0.83	0.65	0.66	0.81	0.81	0.67	0.66
	MobileNetV2 (3D)	0.74	0.73	0.80	0.78	0.67	0.67	0.77	0.77	0.70	0.70
	ShuffleNetV2 (2D)	0.74	0.74	0.78	0.77	0.66	0.66	0.78	0.78	0.67	0.66
	MobileNetV2 (2D)	0.73	0.72	0.75	0.74	0.65	0.65	0.72	0.72	0.68	0.69
**All samples (GE + Siemens + Philips)**	ShuffleNetV2 (3D)	0.78	0.78	0.80	0.80	0.70	0.70	0.80	0.78	0.67	0.65
	MobileNetV2 (3D)	0.74	0.74	0.79	0.78	0.68	0.68	0.76	0.73	0.71	0.71
	ShuffleNetV2 (2D)	0.78	0.78	0.77	0.78	0.71	0.72	0.76	0.76	0.66	0.65
	MobileNetV2 (2D)	0.70	0.70	0.72	0.72	0.68	0.67	0.74	0.75	0.68	0.68
**One-third samples(GE + Siemens + Philips)**	ShuffleNetV2 (3D)	0.72	0.73	0.75	0.75	0.66	0.65	0.75	0.75	0.63	0.60
	MobileNetV2 (3D)	0.70	0.68	0.75	0.75	0.67	0.67	0.72	0.70	0.67	0.67
	ShuffleNetV2 (2D)	0.74	0.73	0.75	0.75	0.67	0.67	0.73	0.72	0.62	0.62
	MobileNetV2 (2D)	0.67	0.67	0.68	0.68	0.64	0.65	0.71	0.70	0.65	0.66

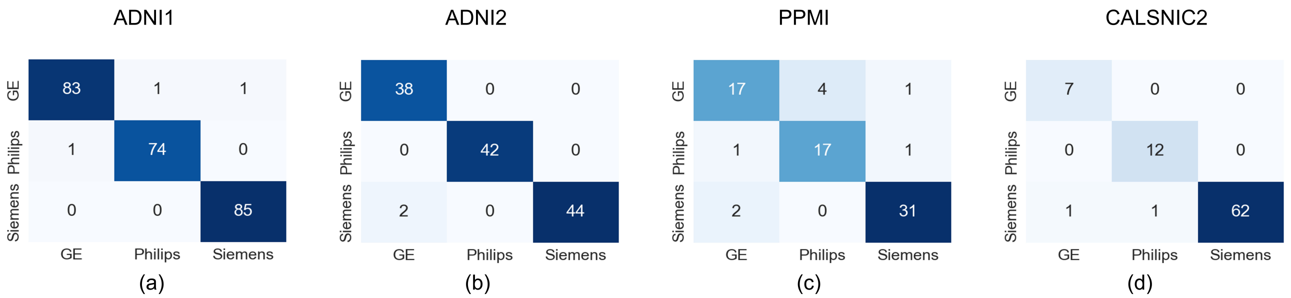

Figure S1: MRI scanner manufacturer classification results for the ADNI1, ADNI2, PPMI, and CALSNIC2 datasets generated by ShuffleNetV2 model. The classification accuracy is approximately 99% for the (a) ADNI1, (b) ADNI2, and (d) CALSNIC2 datasets whereas the accuracy is around 94% for the (c) PPMI dataset.

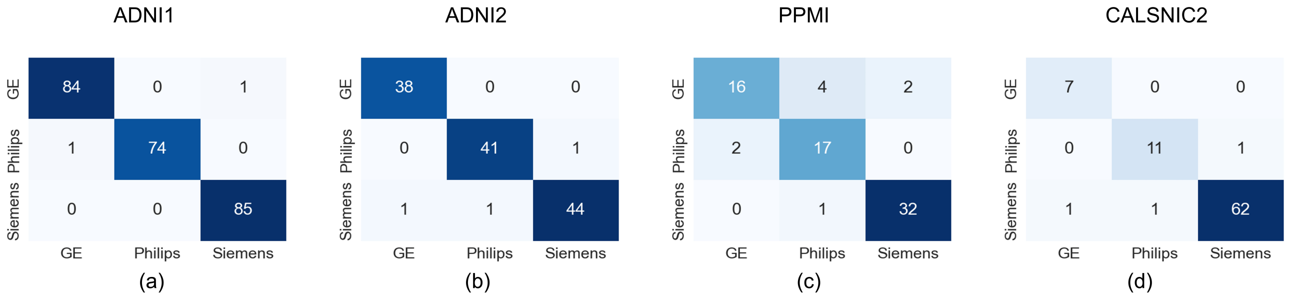

Figure S2: MRI scanner manufacturer classification results for the ADNI1, ADNI2, PPMI, and CALSNIC2 datasets generated by MobileNetV2 model. The classification accuracy is approximately 98% for the (a) ADNI1, (b) ADNI2, and (d) CALSNIC2 datasets whereas the accuracy is around 94% for the (c) PPMI dataset.

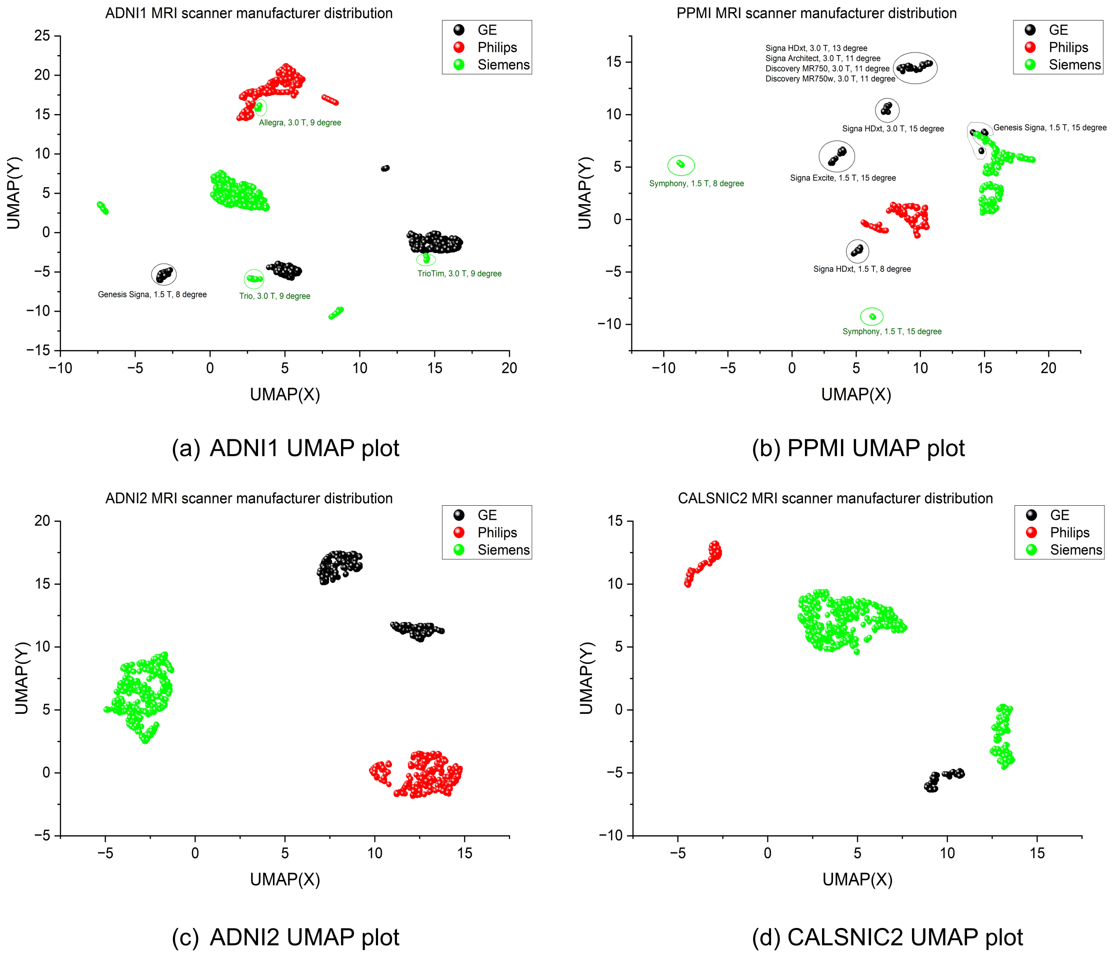

Figure S3: UMAP plots for the ADNI1, ADNI2, PPMI, and CALSNIC2 datasets using the features generated by MRQy evaluation metrics. Different clusters are primarily formed based on the scanner manufacturer. In panels (a) and (b), bounding boxes are delineated, incorporating information about the scanner model, field strength, and flip angle. These annotations visually highlight their role in inducing domain shift within a dataset.

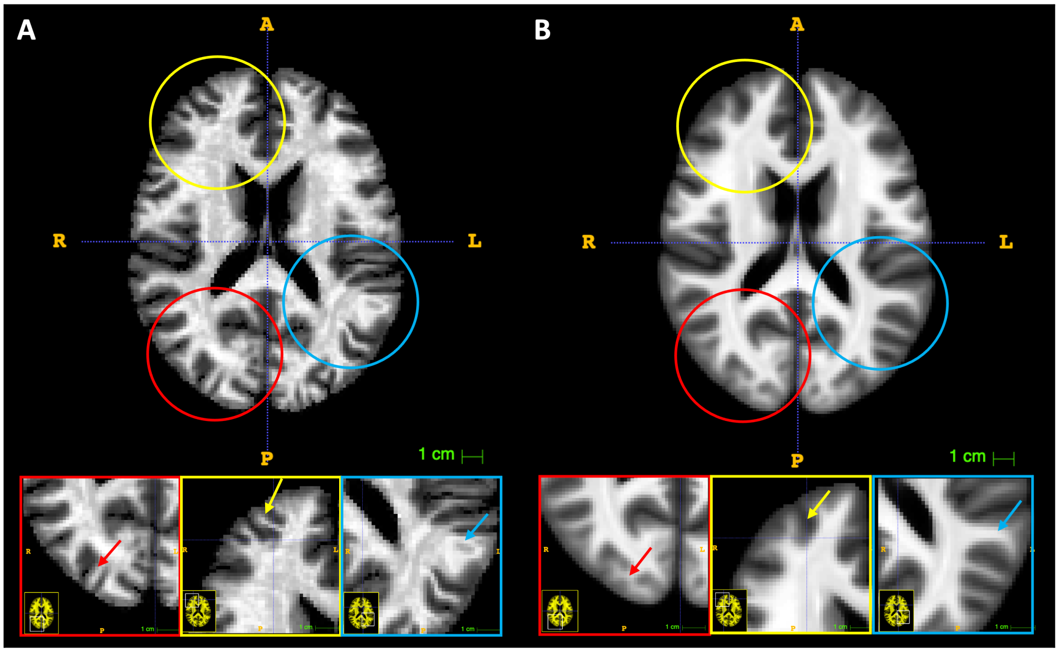

Figure S4: Undesirable effects in voxel-wise ComBat-GAM harmonization of structural MRI. A) One 2D axial slice of 3D MR image of CALSNIC2 dataset before harmonization, B) corresponding slice after harmonization. The red, yellow, and blue arrows point to the regions with manipulated structures, including the disappearance of details or abnormal shape changes, resulting from the ComBat-GAM harmonization.
